# Reoccurring Episcleritis and the Role of Antioxidants

**DOI:** 10.7759/cureus.24111

**Published:** 2022-04-13

**Authors:** Lokesh Goyal, Kunal Ajmera, Ramesh Pandit

**Affiliations:** 1 Hospital Medicine, Christus Spohn, Corpus Christi, USA; 2 Hospital Medicine, Calvert Health Medical Center, Prince Frederick, USA; 3 Internal Medicine, Drexel College of Medicine, Philadelphia, USA; 4 Hospital Medicine, Crozer Chester Medical Center, Upland, USA

**Keywords:** eye discharge, eye pain, vitamin - c, inflammation of eye, episcleritis

## Abstract

Episcleritis is an irritation of the episclera of our eyes. Episclera is a thin layer of tissue present between the conjunctiva and sclera. There are mainly four causes of episcleritis: an allergic response, autoimmune, vascular disease, or infections. The symptoms are redness, erythema, discomfort in the eyes, and clear discharge. It can sometimes be painful as well. In this case presentation, we will discuss the role of vitamin c in the prevention and reoccurrence of autoimmune episcleritis.

## Introduction

Most cases of episcleritis are idiopathic and usually resolve on their own. However, the rest of the cases of episcleritis are usually associated with systemic disorders, for example, rheumatoid arthritis, Crohn's disease, systemic lupus erythematosus, etc. There are some infectious causes as well associated with episcleritis, such as syphilis, Lyme disease, herpes virus, etc., but they are less common when compared to systemic and vascular disorders [1-5].

## Case presentation

The patient is a 60-year-old male who presents to my outpatient clinic with a chief complaint of redness, irritation, and watery eyes. He states that this has happened multiple times in the past since the age of 45 years. The patient states that the symptoms are abrupt, and they typically take about one to two weeks to resolve. He has tried multiple medications, including steroids, NSAIDs, etc., which work, but these symptoms always return back in two to three months. He has a past medical history only significant for hypertension for which he takes amlodipine 5 mg daily. He does not smoke, does not drink, and has never used any drugs. He has normal vision and does not use spectacles. The patient's family history is only significant for coronary artery disease in both parents. He has a desk job in a bank and is not exposed to any chemicals. The patient saw an ophthalmologist two years ago for inflammation of his eyes. He was referred to a rheumatologist because of recurring inflammation of the eyes. He had extensive lab work done, which included checking for autoimmune diseases like systemic lupus erythematosus, Sjögren's syndrome, rheumatoid arthritis, etc., which were all negative. The patient was also tested for herpes zoster and Lyme disease, which were negative. He even had a chest x-ray and CT chest performed by his rheumatologist, which all turned out to be negative. The patient states that he does not take any supplements. He has tried taking herbal supplements like honey to help with his eye, but they have never worked. He occasionally uses over-the-counter artificial tear drops to prevent recurring inflammation of his eye, but it does not help. The patient changed his soap and shampoo and relocated to a new city over the past 15 years (since his symptoms started) but has had no relief in his symptoms.

**Figure 1:**
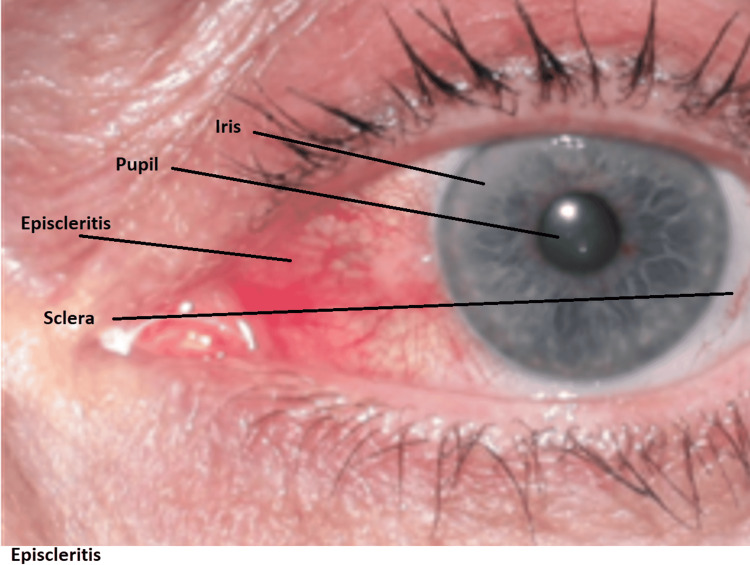
Red episclera and slight swelling of the episclera without any swelling or edema in the sclera

A physical exam reveals red episclera and slight swelling of the episclera without any swelling or edema in the sclera, as shown in Figure 1. The patient underwent laboratory evaluations and results in Table 1.

**Table 1:** Laboratory evaluations and results

Labs Test	Results	Normal Range
Complete Blood Count (CBC)	All within normal range	Normal
Comprehensive Metabolic Panel (CMP)	All within normal range	Normal
Rheumatoid Factor	Negative	Negative
Antinuclear Antibody	negative	Negative
Urinary analysis	Negative	Negative
Thyroid-Stimulating Hormone (TSH)	1	0.35-5.0

He was started on over-the-counter artificial tears and oral Ibuprofen - 400mg three times daily for seven days which resolved the patient's symptoms. However, the patient returned to my clinic again in two months with the same symptoms. Because of the patient's recurrence and multiple episodes in the past, I prescribed the same dose of Ibuprofen, but this time I also prescribed 500mg of Vitamin C daily. I advised the patient to continue taking Vitamin C daily for the rest of his life even after his symptoms completely resolved. The patient returned to me after seven months for his regular physical exam and stated that since starting his daily dose of Vitamin C, he had not had any more episodes of episcleritis.

## Discussion

The immune system of our body can eventually become dysregulated during our lifecycle, which causes autoimmune attacks on parts of our body, for example, Inflammatory bowel disease, Hashimoto's disease, juvenile arthritis, etc. Similarly, the eyes can also be affected, leading to diseases like episcleritis. Studies have shown an association between oxidative stress and inflammation. Inflammation causes an increase in oxidative stress by elevating reactive oxygen species (ROS) [6,7]. Increased oxidative stress and ROS have been associated with inflammatory responses and autoimmune diseases, including keratoconjunctivitis sicca [8,9].

As we know, Vitamin C is a strong antioxidant that protects our tissue from oxidative damage. The total amount of Vitamin C in the human retina is 20 times higher than in the plasma [8]. Other Vitamins, which are also antioxidants, include Vitamin A and E. In autoimmune episcleritis, we suspect that the antioxidant system somehow gets weak, which causes damage to the episclera and causes inflammation and disease [8]. Vitamin C deficiency has been known to be associated with corneal inflammation, with some patients presenting with dry, irritated eyes [10]. Vitamin C supplementation can also be beneficial in undiagnosed cases of deficiency, as well as for its role in reducing oxidative stress, thereby resolving recurrent symptoms [11].

Regular Vitamin C intake is associated with minimal side effects, even at high intravascular doses [12]. There have been reports of kidney stones or other complications in patients with glucose-6-phosphate dehydrogenase deficiency or those with poor renal function receiving supraphysiological doses of vitamin C, and caution is recommended in such clinical situations [13,14]. These adverse effects could be minimized with patient education and by limiting daily intake within the upper limits of daily intake of 2000mg/day for adults [15].

## Conclusions

Episclera is a highly vascular tissue that lies above the sclera and beneath the conjunctiva. Episcleritis is an inflammatory response likely caused by oxidative stress, which causes tissue inflammation. In this case, increasing the effectiveness of our body's antioxidant system could contain inflammation and disease. There are different kinds of antioxidants available in our diet and also available as synthetic supplements such as Vitamin C, which has been shown to prevent ocular inflammation. We conclude that Vitamin C can be considered a preventive and therapeutic measure in patients with recurrent episcleritis, which also has an excellent safety profile. Still, additional rigorous investigations with case-controlled studies or trials are needed for a more definitive conclusion.
